# Self and Non-self Discrimination of Intracellular Membranes by the Innate Immune System

**DOI:** 10.1371/journal.ppat.1003538

**Published:** 2013-09-19

**Authors:** Jörn Coers

**Affiliations:** Departments of Molecular Genetics and Microbiology and Immunology, Duke University Medical Center, Durham, North Carolina, United States of America; The University of North Carolina at Chapel Hill, United States of America

## Overview

To discriminate self from non-self, the innate immune system evolved a large repertoire of germline-encoded receptors that detect molecular patterns associated with infections [1]. As originally proposed by the late Charles Janeway, such pattern recognition receptors (PRRs) [2] detect three broadly defined categories of patterns that can best be described as “microbial non-self,” “induced- or aberrant-self,” and “missing-self” [3]. The minimal definition of each pattern consists of two terms: 1) the nature of the PRR ligand(s) and 2) its cellular context, especially its precise location within the infected organism [4]. For example, the bacterial non-self molecule lipopolysaccharide is immunostimulatory in the intestinal crypts but typically not in the gut lumen [5]. Similarly, a number of intracellular self molecules including ATP or DNA-binding proteins activate PRRs and elicit immune responses exclusively when released from infected or injured cells into the extracellular milieu [6], [7]. Therefore, in order to detect molecular patterns, the expression of PRRs must be and indeed is spatially compartmentalized [1]. Accordingly, PRRs are traditionally defined by the location of their cognate ligands and placed into two separates groups: PRRs for extracellular-derived ligands and PRRs for soluble, cytosolic ligands [1], [4]. Here, I propose the classification of a third group of PRRs that act as intracellular “membrane sensors” by sampling and recognizing properties of intracellular membranes and microbial surfaces that are found inside infected host cells. Although the principles of immune recognition by “membrane-sensing” PRRs as outlined here are most likely widely applicable to intracellular infections with various classes of pathogens, for the sake of simplicity, I will focus on bacterial infections in this *PLOS* Pearls article.

## The Innate Immune System Recognizes and “Marks” Pathogen-Containing Vacuolar Membranes and Cytosolic Bacteria for Autophagic Destruction

Intracellular bacterial pathogens reside and replicate either within the cytosol or inside membrane-bound, pathogen-containing vacuoles (PVs) [8], [9]. To restrict intracellular microbial growth and at the same time prevent damage to the host cell itself, most cell-autonomous host defense mechanisms operate within the boundaries of vacuolar compartments that include phagosomes and lysosomes [8], [10]–[12]. Therefore, the capture of cytosolic microbes as well as PVs inside host-controlled compartments constitutes a critical step in cell-autonomous immunity. Additionally, the host can deliver antimicrobial agents directly to PVs [13], which are already spatially defined by their surrounding membranes. In either case, the host cell must be able to recognize cytosolic bacteria and PVs as *macromolecular, non-self structures* in order to mark them as targets for innate immune responses. Once bacteria or PVs are marked as aberrant or non-self structures, they can be delivered to microbicidal lysosomes, a process that frequently involves the autophagic machinery [8], [10], [12].

Whereas the importance of autophagy in cell-autonomous host defense is well established, we are only beginning to understand the mechanisms by which the host can specifically recognize invading pathogens and PVs as non-self structures. In the following I give three examples describing how the innate immune system can locate intracellular pathogens. Each example highlights one of the three basic types of pattern associated with intracellular pathogens and recognized by the innate immune system, namely the non-self, the aberrant-self, and the missing-self.

## Recognition of Non-self

One marker that labels cytosolic bacteria and PVs as substrates for defense pathways is the small protein ubiquitin [8], [10], [12]. Ubiquitination of microbial invaders and/or their surrounding vacuoles allows the host cells to deliver ubiquitin-coated pathogens to autophagosomes, which can subsequently mature into degradative autolysosomes [8], [10], [12]. Ubiquitination requires a tripartite complex consisting of E1, E2, and E3 enzymes. The highly variable E3 component is pivotal in providing substrate specificity [14]. The groundbreaking discovery that host cells can label intracellular bacteria with ubiquitin [15] raised the question as to which E3 ubiquitin ligase(s) were involved in this process. Recently, Xavier and colleagues found that the E3 ligase LRSAM1 colocalizes with intracellular bacteria and is required for the ubiquitination and autophagic degradation of *Salmonella enterica* serovar *Typhimurium* [16], [17]. LRSAM1 was shown to specifically target the small fraction of cytosolic *S. Typhimurium* that had exited from the protective surroundings of *Salmonella*-containing vacuoles (SCVs).

How is LRSAM1 able to detect cytosolic bacteria? The LRSAM1 protein contains a leucine-rich repeat (LRR) domain, a common feature of PRRs that is often instrumental in the direct binding of microbe-derived molecules by PRRs [1]. Because LRSAM1 can bind directly to bacteria *in vitro* through its LRR domain [16], non-self molecules on the bacterial surface are the most likely candidates to facilitate this binding reaction. Once docked to bacteria, LRSAM1 ubiquitinates itself and possibly also LRSAM1-bound bacteria [16]. The ubiquitinated LRSAM1-bacteria complex is subsequently captured and degraded inside autolysosomes [16], [17]—a process that is sometimes referred to as xenophagy [12].

LRSAM1 represents the first member of what will most likely emerge as a larger group of cytosolic PRRs that bind directly to non-self ligands decorating microbial cell surfaces. As effective as these LRSAM1-like PRRs may turn out to be in fighting cytosolic pathogens, they cannot provide protection against pathogens residing within the confines of PVs. Therefore, in order to locate pathogens hidden inside vacuoles, the innate immune system must be able to detect additional patterns that discriminate non-self PVs from self vacuoles.

## Recognition of Aberrant-Self

Intracellular bacterial pathogens have developed complex mechanisms to create and maintain PVs as their intracellular residence [9]. To do so, vacuolar pathogens access the cytosol through protein secretion systems and membrane toxins. These various interactions between vacuolar pathogens and their surroundings can result in damaged PV membranes [4]. Recent work by Randow and colleagues demonstrated that damaged membranes of SCVs are sensed by cytosolic lectins of the galectin family [18].

Galectins constitute a family of β-galactoside–binding proteins, which exist as transmembrane, secreted, and cytosolic forms [19]. Galectins can bind to non-self glycans coating the surface of parasites as well as to self glycans found on the extracellular leaflet of the mammalian plasma membrane and the luminal side of intracellular vacuoles [19]. If intracellular vacuoles, e.g., PVs, are damaged and lose their membrane integrity, luminal glycans become exposed to the cytosol. Therefore, glycans interfacing with the cytosol represent an aberrant-self pattern that can be detected by cytosolic galectins [18], [20], [21]. This pattern occurs, for example, when intracellular bacterial pathogens escape from their vacuoles to enter the cytosol [21]. Accordingly, galectins have been shown to colocalize with the vacuolar remnants left behind by “professional” cytosolic pathogens like *Shigella flexneri* and *Listeria monocytogenes* [20], [21]. However, galectins can also target “professional” vacuolar pathogens like *Salmonella*. One galectin specifically, Galectin-8, has been shown to translocate to disrupted SCVs and to recruit the autophagy adaptor protein NDP52 to SCVs, thereby delivering *S. Typhimurium* for degradation inside autolysosomes [18].

Because loss of membrane integrity is an aberrant-self pattern that can be recognized by the innate immune system, vacuolar pathogens must have evolved mechanisms to either inhibit detection or prevent the occurrence of the pattern itself. The latter strategy is indeed employed by at least two bacterial pathogens, namely by *Legionella pneumophila* and *S. Typhimurium*. Both of these pathogens secrete specific bacterial effector proteins into the host cytosol to prevent PV disruption [22], [23], albeit by incompletely understood mechanisms. The specific *Salmonella* effector protein SifA is required to maintain SCV membranes [22]. Salmonella *sifA* mutants colocalize with galectins at significantly higher rates than coisogenic wild-type bacteria [21], illustrating that the maintenance of PV membrane integrity is an important mechanism by which vacuolar pathogens avoid immune detection.

## Recognition of Missing-Self

Pathogens invest considerable resources into preserving the stability of their surrounding vacuoles. For example, the obligate intracellular pathogen *Chlamydia trachomatis* forms rigid cages made out of actin and intermediate filaments to stabilize its vacuole [24], the so-called “inclusion.” This example illustrates that, more often than not, a given PV will lack aberrant-self patterns that result from deteriorating PV membranes. Therefore, to locate intact PVs, the innate immune system must be able to detect patterns that are unrelated to diminishing vacuolar stability. We have recently demonstrated that the host cell marks self membranes with a set of “guard” proteins called IRGMs and that the innate immune system can identify PVs as non-self due to the “missing” of IRGM guards from PVs [25].

**Figure 1:**
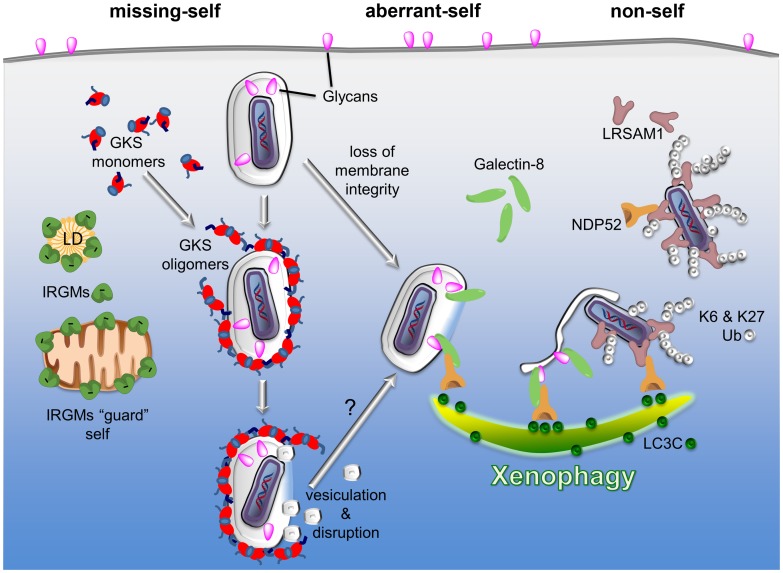
PRRs detect three types of patterns associated with the macromolecular structure of cytosolic bacteria and PVs: missing-self, aberrant-self, and non-self. Three examples of PRRs are given to highlight the principles by which the innate immune system discriminates self membranes from PVs and cytosolic bacteria. I) Missing-self: in order to bind to membranes, GKS proteins of the IRG family of GTPases must first exchange GDP for GTP. In the GTP-bound, “activated” state, GKS proteins oligomerize and stably associate with membranes. IRGM proteins residing on self membranes, e.g., on lipid droplets (LDs) or mitochondria, inhibit GKS activation and thus shield self membranes against GKS proteins. PVs formed by microbes like *C. trachomatis* are devoid of IRGM proteins and therefore permissive for GKS protein binding. II) Aberrant-self: host-derived glycans decorate the plasma membrane as well as the luminal side of vacuoles, including PVs. In disrupted vacuoles, glycans become exposed to the cytosol. Cytosolic Galectin-8 can bind to exposed glycans and subsequently recruit the autophagy adaptor protein NDP52 to these “broken” vacuoles. III) Non-self: the ubiquitin ligase LRSAM1 can directly bind to bacterial surfaces. Once attached to cytosolic bacteria, LRSAM1 polyubiquitinates itself, forming predominantly K6 and K27 linkages. Additionally, LRSAM1 binds to NDP52 to promote autophagic degradation of cytosolic bacteria (xenophagy). Although the three PRRs listed here detect distinct patterns, they are likely to cooperate in immune surveillance. For example, GKS proteins rupture PV membranes and thus induce an aberrant-self pattern in PVs that is recognizable by galectins. Subsequent recruitment of autophagy adaptor proteins may further promote the disintegration of PV membranes, thereby allowing LRSAM1 to bind directly to the disrobed microbe.

IRGM proteins belong to a family of immunity-related GTPases (IRGs) [26]. Expression of IRG proteins is induced by pro-inflammatory interferons (IFNs). The IRG protein family can be divided into two groups: the aforementioned membrane-bound IRGM proteins and the predominantly cytosolic GKS proteins [26]. Once an IFN-activated mouse cell is infected with the human pathogen *C. trachomatis*, cytosolic GKS proteins translocate to *C. trachomatis* inclusions [27], and eliminate inclusions, in all likelihood, by inducing vacuolar rupture [28]. The raptured inclusions and/or the released bacteria subsequently become targets for autophagic clearance [29], [30], a process that may involve additional membrane-sensing PRRs (see Figure 1).

In order to bind to membranes, GKS proteins must transition into the “active” state by exchanging GDP for GTP [31]. Because IRGM proteins act as guanine dissociation inhibitors of GKS proteins [31], IRGM-decorated endomembranes minimize the amount of active GKS proteins in their vicinity and are therefore protected against GKS binding [25]. *C. trachomatis* inclusions on the other hand are missing substantial amounts of IRGM proteins and are therefore unable to block GKS protein activation and membrane binding [25]. While the reasons for the absence of IRGM from *C. trachomatis* inclusions remains mysterious, our studies show that the lack of endogenous IRGM proteins serves as a missing-self pattern associated with a subset of PVs [25].

## Conclusion

To direct cell-autonomous defenses specifically to cytosolic bacteria and PVs, the host cell must be able to discriminate intracellular self membranes from non-self membranes. Only very recently have we begun to identify membrane-sensing PRRs that can survey the vesicular landscape of a mammalian cell: this class of PRRs includes LRSAM1, galectins, and IFN-inducible GTPases. How these PRRs interact with the network of immune surveillance and how they orchestrate innate as well as adaptive immune responses are important, unanswered questions. Finding answers to these questions will vehemently advance our understanding of innate immunity and infectious disease.
